# The role of multispecies social interactions in shaping *Pseudomonas aeruginosa* pathogenicity in the cystic fibrosis lung

**DOI:** 10.1093/femsle/fnx128

**Published:** 2017-07-13

**Authors:** Siobhán O'Brien, Joanne L. Fothergill

**Affiliations:** 1 Center for Adaptation to a Changing Environment (ACE), ETH Zürich, 8092 Zürich, Switzerland; 2 Department of Biology, University of York, Wentworth Way, York YO10 5DD, UK; 3 Institute of Infection and Global Health, University of Liverpool, 8 West Derby Street, Liverpool L69 7B3, UK

**Keywords:** interspecific interactions: multispecies interactions, microbiome, cystic fibrosis, *Pseudomonas aeruginosa*, microbial communities

## Abstract

*Pseudomonas aeruginosa* is a major pathogen in the lungs of cystic fibrosis (CF) patients. However, it is now recognised that a diverse microbial community exists in the airways comprising aerobic and anaerobic bacteria as well as fungi and viruses. This rich soup of microorganisms provides ample opportunity for interspecies interactions, particularly when considering secreted compounds. Here, we discuss how *P. aeruginosa*-secreted products can have community-wide effects, with the potential to ultimately shape microbial community dynamics within the lung. We focus on three well-studied traits associated with worsening clinical outcome in CF: phenazines, siderophores and biofilm formation, and discuss how secretions can shape interactions between *P. aeruginosa* and other commonly encountered members of the lung microbiome: *Staphylococcus aureus*, the *Burkholderia cepacia* complex, *Candida albicans* and *Aspergillus fumigatus.* These interactions may shape the evolutionary trajectory of *P. aeruginosa* while providing new opportunities for therapeutic exploitation of the CF lung microbiome.

## Teaser

How social interactions among microbes living in the cystic fibrosis lung may be influencing patient health.

## INTRODUCTION

Individuals with cystic fibrosis (CF) suffer from a buildup of thick, viscous mucous in the airways, predisposing them to lifelong bacterial lung infections which are often fatal. *Pseudomonas aeruginosa* is the most common pathogen in CF, displaying high levels of antibiotic resistance and virulence—so that elimination is apparently impossible (Pressler *et al.*^2011^). Chronic infection with *P. aeruginosa* is associated with deterioration of pulmonary function, reduction in quality of life and premature death (Koch and Høiby 1993; Emerson *et al.*^2002^; Hart and Winstanley 2002).

The CF lung airways consist of polymicrobial infections that vary in their composition and diversity throughout a patient's lifetime. Diversity typically increases during the first decade of life, and decreases thereafter (Cox *et al.*^2010^; Klepac-Ceraj *et al.*^2010^). While *Haemophilus influenzae* and *Staphylococcus aureus* are present mainly in young children, by the age of 20, 60–70% of CF patients present intermittent colonisation by *P. aeruginosa* (Folkesson *et al.*^2012^). Earlier acquisition of *P. aeruginosa* has been associated with a more rapid decline in lung function and poorer clinical outcomes (Emerson *et al.*^2002^). In at least 50% of adult CF patients, *P. aeruginosa* has been reported as the dominant organism, displacing the resident microbial community (Valenza *et al.*^2008^). Furthermore, CF patients infected with *P. aeruginosa* are vulnerable to developing secondary infections, for example with the *Burkholderia cepacia* complex, predisposing patients to necrotising pneumonia, which is usually fatal (Sajjan *et al.*^2001^; Bragonzi *et al.*^2012^). Fungi and yeasts also inhabit the airways, where *Aspergillus fumigatus* and *Candida albicans* are the most prevalent fungi and yeast, respectively (Chotirmall and McElvaney 2014). Although their prevalence is likely underestimated and detection methods vary between diagnostic laboratories, both *Aspergillus* spp. and *Candida* spp. have been identified in up to 50% of CF patients (Pihet *et al.*^2009^; Chotirmall *et al.*^2010^).

The recent surge in the number of studies employing in-depth, parallel, next-generation sequencing of CF lung microbial communities has given a greater insight into what exactly lives in this complex ecosystem. Inhabiting microorganisms range from recognised pathogens such as *Pseudomonas* spp. and *Burkholderia* spp. to bacteria less understood in the context of CF such as *Prevotella* spp. and *Veillonella* spp. (Fodor *et al.*^2012^; Boutin *et al.*^2015^), and classically commensal microorganisms such as oral streptococci. A novel isolation method led to the detection of *Candida dubliniensis* in patients >30 years old with advanced stages of the disease, although the importance of this fungal pathogen in CF is not yet understood (Sahand *et al*. 2006; Chortimall *et al.*^2010^). Lower respiratory tract microbiome studies have also supported the identification of new proposed pathogens in the CF lung such as *Ralstonia mannitolilytica*, identified in seven patients in Canada and associated with accelerated disease progression and raised mortality (Coman *et al.*^2017^). In addition to identifying novel bacterial species, metagenomic studies have revealed a diverse viral community in the CF lung with over 450 viral genotypes identified (Lim *et al.*^2014^). Furthermore, some of these viruses have been linked to the onset of pulmonary exacerbations (periods of acute worsening of pulmonary symptoms) (Billard *et al.*^2017^).

Lung microbial diversity tends to decrease with increasing disease severity (as *P. aeruginosa* dominates the population) (Cox *et al.*^2010^; Fodor *et al.*^2012^; Frayman *et al.*^2017^). However, whether this association is linked to increased *P. aeruginosa* pathogenicity remains elusive. Lung community diversity can be highly patient specific and no universal indicator of the onset of exacerbation has been identified so far (Whelan *et al.*^2017^). Furthermore, during antibiotic treatment, limited changes in microbial community structure have been identified (Fodor *et al.*^2012^; Li *et al.*^2016^).

Through our progressive understanding of the complexities of polymicrobial communities, it is becoming increasingly clear that interactions between bacterial pathogens and the microbial community within which they reside can influence pathogenesis, antimicrobial resistance and disease progression (Hoffman *et al.*^2006^; Peters *et al.*^2012^; Antonic *et al.*^2013^; Baldan *et al.*^2014^; Fugère *et al.*^2014^; Beaume *et al.*^2015^). However, it is often difficult to elucidate whether these clinical changes are a cause or consequence of these interactions. In this review, we highlight the role of multispecies interactions in shaping *P. aeruginosa* virulence, and discuss examples where these interactions may be of paramount importance in predicting patient health. Secreted products by *P. aeruginosa* are likely to influence neighbouring microorganisms, and it is reasonable to suggest that community context may in turn shape the relative costs and benefits associated with these secretions. Crucially, this implies that the role of some CF microorganisms in disease may be subtle, acting through cross-species interactions rather than being recognised pathogens *per se.*

## HOW MIGHT MULTISPECIES INTERACTIONS SHAPE *P. aeruginosa* VIRULENCE?

Over the course of chronic infections*, P. aeruginosa* CF isolates commonly display adaptive phenotypes such as conversion to mucoidy and loss of motility, as well as reduced expression of acute virulence factors and extracellular toxins (Smith *et al.*^2006^; Bragonzi *et al.*^2009^; Folkesson *et al.*^2012^; Lorè *et al.*^2012^; Davies *et al.*^2016^; Winstanley, O’Brien and Brockhurst 2016). Despite the general trend toward loss of virulence as *P. aeruginosa* becomes chronic, it is becoming increasingly clear that loss of virulence is not universal within a patient. Furthermore, *P. aeruginosa* isolates within patients are typically highly diverse with respect to the aforementioned phenotypic characteristics (Fothergill *et al.*^2010^; Mowat *et al.*^2011^; O’Brien *et al.*^2017^). Despite the potential for *P. aeruginosa* adaptive evolution to influence patient health, both the causes and consequences of these adaptive changes are not well understood. The ability of many microbial secretions to influence the fitness of other organisms either directly (e.g. bacteriocin-mediated killing) or indirectly (e.g. antibiotic degradation), with potential for positive (cooperation) or negative (competition) fitness consequences, suggests that microbial interactions may play an integral role in shaping *P. aeruginosa* evolution within the CF lung.

Here, we focus on four clinically relevant *P. aeruginosa* traits that may, in part, shape and be shaped by interactions with the natural microbial community. Crucially, these traits have potential to be ‘social’—that is, they may directly or indirectly influence the fitness of nearby cells (West *et al.*^2007^). This list is not exhaustive, but should be regarded as examples of microbial traits whose role cannot be fully understood without consideration of community context.

### Phenazine production

**Figure 1.**
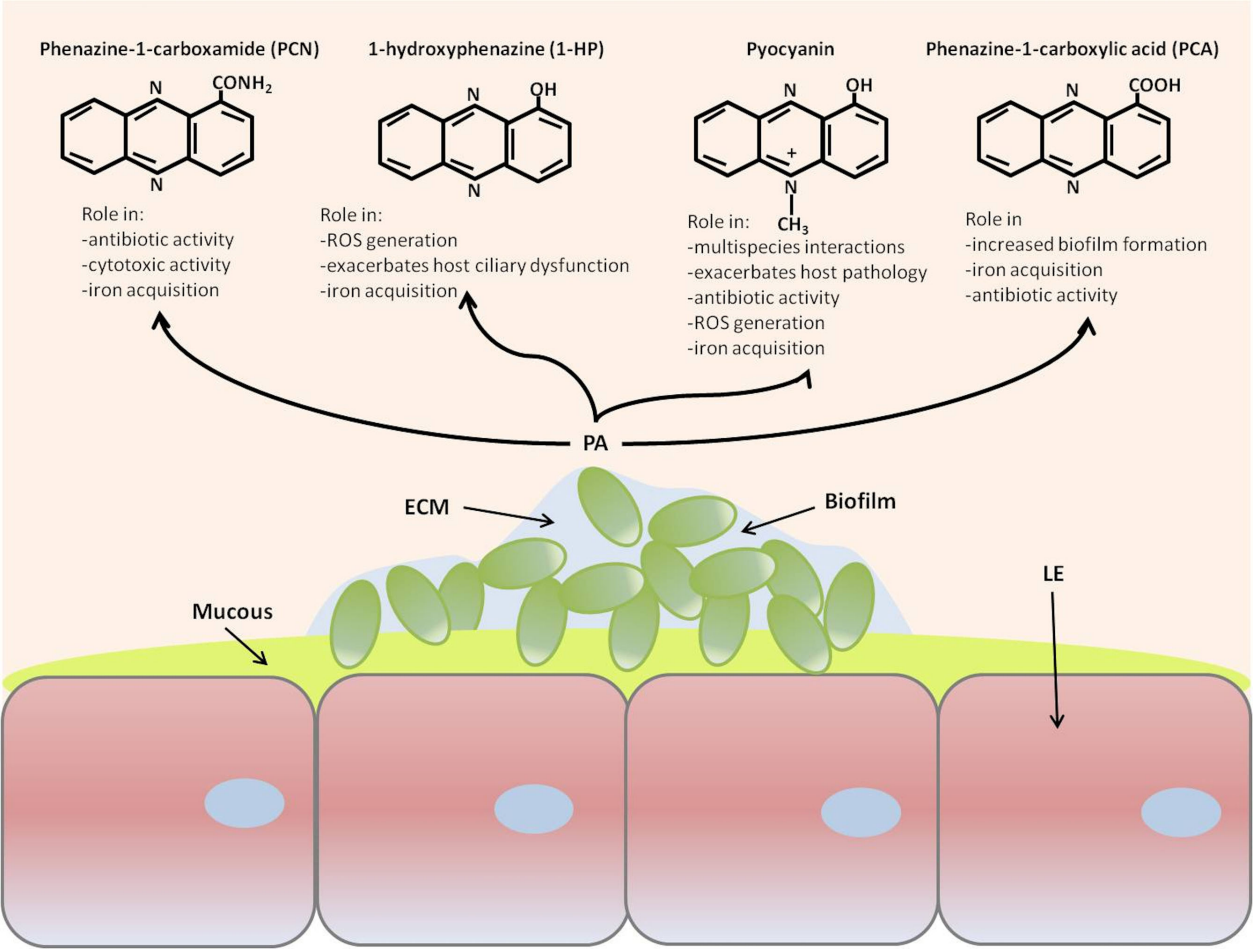
Schematic representation of phenazine production by *Pseudomonas aeruginosa* growing in a biofilm in the cystic fibrosis lung. PA = *P. aeruginosa*, ECM = extracellular matrix, LE = lung epithelium.

Phenazines are secondary metabolites produced by a variety of bacteria, notable for their broad-spectrum antibiotic properties and roles in virulence (Sorensen and Klinger 1987). Phenazine production is mediated by quorum sensing (QS), a method of bacterial cell–cell communication that allows the coordinated expression of genes in bacterial populations (Dietrich *et al.*^2006^). *P. aeruginosa* secretes four main classes of phenazines: pyocyanin, phenazine-1-carboxamide (PCN), 1-hydroxyphenazine (1-HP) and phenazine-1-carboxylic acid (PCA) (Fig. 1). One class of phenazine, pyocyanin, is a blue redox-active pigment that exerts a host inflammatory response, impairs ciliary function and induces oxidative stress within the lung (O’Malley *et al.*^2003^; Winstanley and Fothergill 2009). While the effects of pyocyanin on the host may influence other microorganisms indirectly, there is some evidence that pyocyanin can also have a direct role in shaping microbial communities. Pyocyanin can function as an iron-reducing agent, allowing iron-limited microorganisms to thrive (see below) (Cox 1986). Furthermore, the bactericidal effect of pyocyanin may reduce community diversity (Norman *et al.*^2004^) and select for a community of resistant species. Two recent studies (Korgaonkar and Whiteley 2011; Korgaonka *et al.*^2013^) reported that *P. aeruginosa* responds directly to cell wall fragments from Gram-positive bacteria by increasing production of multiple extracellular factors, including pyocyanin. Co-infection of *P. aeruginosa* with avirulent Gram-positive bacteria in both rat lung and *Drosophila* models resulted in increased lung damage and overall enhanced virulence, respectively (Duan *et al.*^2003^; Korgaonka *et al.*^2013^), although the exact mechanisms are unknown. Clinical isolates respond similarly: Whiley *et al.* (2014) reported enhanced *P. aeruginosa* pyocyanin production when co-cultured with oral viridans streptococci (*Streptococcus oralis*, *Streptococcus mitis*, *Streptococcus gordonii* and *Streptococcus sanguinis*), and these co-cultures exhibited increased pathogenicity in an insect host model compared with *P. aeruginosa* alone. However, in this case increased pathogenicity might also have arisen from other virulence-associated secretions, rather than pyocyanin *per se*.

Studies in which animal models are infected with *P. aeruginosa* strains producing varying levels of pyocyanin reveal that pyocyanin production tends to lead to more virulent infections (Mahajan-Miklos *et al.*^1999^; Cao, Baldini and Rahme 2001; Lau *et al.*2004a,b; Courtney *et al.*^2007^; O’Brien *et al.*^2017^). In CF, periods of patient exacerbations have been linked with increased pyocyanin production in the lung (Fothergill *et al.*^2007^, ^2010^; Mowat *et al.*^2011^). However, not all patients with worsening symptoms harbour increased numbers of overproducing phenotypes (Nguyen and Singh 2006; Smith *et al.*^2006^), and the causality of this relationship remains unconvincing. Furthermore, why pyocyanin overproducers evolve and thrive in some scenarios and not others remains to be elucidated. Interestingly, while virulence is predictably lost over the course of CF infections, longitudinal studies of pyocyanin production have so far failed to detect any predictable evolutionary changes over the course of chronic infections (Jiricny *et al.*^2014^; Winstanley, O’Brien and Brockhurst 2016). We speculate that multispecies interactions can at least partly explain the observed fluctuations in pyocyanin production. If this is the case, assays for pyocyanin production by clinical isolates in media or even artificial sputum models that mimic abiotic conditions in the CF lung (e.g. Fothergill *et al.*^2010^; Mowat *et al.*^2011^; Jiricny *et al.*^2014^; O’Brien *et al.*^2017^) may not be sufficient indicators of what these strains are producing *in vivo*. Ultimately, by understanding whether community context matters for *P. aeruginosa* pyocyanin production, it may be possible to manipulate the lung microbiome to reduce the severity of clinical symptoms during CF-associated exacerbations.

### Biofilm formation

**Figure 2.**
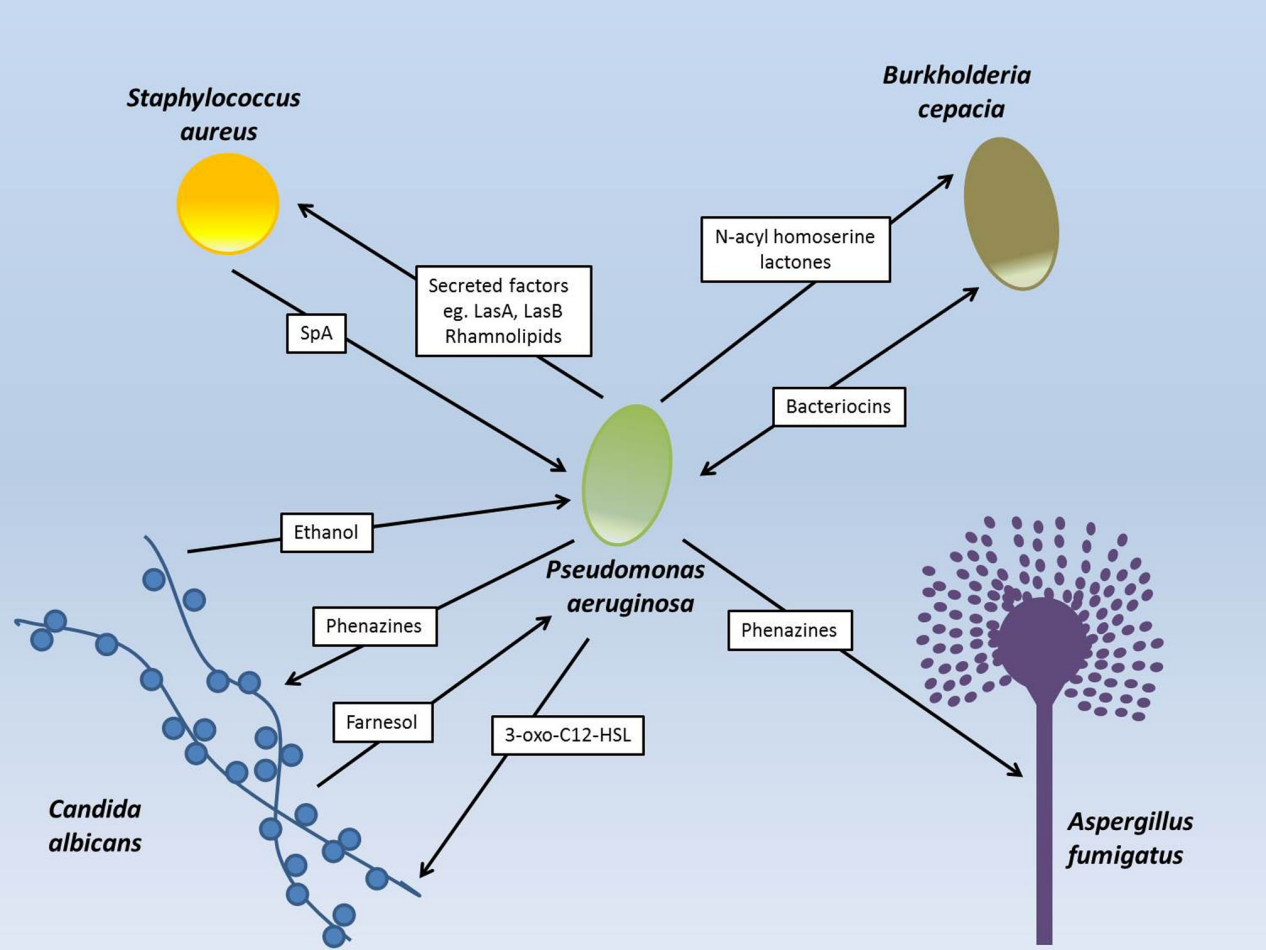
Summary of discussed interactions between *Pseudomonas aeruginosa* and other microbial inhabitants of the cystic fibrosis lung. Arrows depict the direction of the interaction. Note that we have omitted interactions driven by iron acquisition in this figure because the ability of siderophores to shape interactions is likely to be driven mainly by indirect effects of iron limitation.

The intractability of *P. aeruginosa* in CF has been largely attributed to the presence of mucoid alginate-producing strains in the later stages of infection (Ramsey and Wozniak 2005; Sousa and Periera 2014; Winstanley, O’Brien and Brockhurst 2016). These strains form resilient biofilms, conferring enhanced resistance to antibiotics, phage and the host immune system, ultimately causing a decline in lung function (Høiby *et al.*^2010^; Høiby, Ciofu and Bjarnsholt 2010). While this transition to mucoidy is commonly viewed as a global response to environmental stress (e.g. Davies *et al.*^2016^), there is some evidence that multispecies social interactions may play a role. For instance, ethanol produced by *C. albicans* stimulates biofilm formation in *P. aeruginosa* (DeVault, Kimbara and Chakrabarty 1990), while a protein secreted by *S. aureus*, SpA, inhibits it (Armbruster *et al.*^2016^) (Fig. 2). Exopolysachharides can also impact on spatial organisation in polymicrobial biofilms (Chew *et al.*^2014^). One *P. aeruginosa* exopolysaccharide, Pel, is required for a close association in biofilms with *S. aureus*. However, another exopolysaccharide, Psl, allows *P. aeruginosa* to form a single species biofilm on top of *S. aureus.* Therefore, the type of exopolysaccharide produced by *P. aeruginosa* can impact the architecture of the biofilm and the ability of these two species to interact closely (Chew *et al.*^2014^).

Viruses of bacteria (phages) have also been described in the CF lung (Lim *et al.*^2013^), and are a promising novel way of eliminating drug-resistant pathogens (Waters *et al.*^2017^). Interactions between *P. aeruginosa* and lytic phages (which lyse the bacterial cell upon infection) may drive the transition to mucoidy by enhancing resistance to phage infection (Miller and Rubero 1984; Scanlan and Buckling 2012). Conversely, evolving *P. aeruginosa* with temperate phages (which can either complete the lytic cycle or integrate into the bacterial chromosome as a prophage), can reduce biofilm formation by accelerating the loss of biofilm-dependent type IV pili (Davies *et al.*^2016^). While understanding how the abiotic and biotic environment interact to promote mucoidy is no easy task, it is an endeavour worth pursuing. Mucoid variants of *P. aeruginosa* are highly problematic in the clinic, and novel therapeutics aimed at disrupting mucoidy are highly sought after (Romling and Balsalobre 2012; Gnanadhas *et al.*2015a,b).

### Iron-acquisition

Iron is an essential nutrient for many microorganisms, yet in the early stages of CF lung infection the availability of iron for inhabiting microbiota is highly restricted (Tyrrell and Callaghan 2016). *P. aeruginosa* can overcome this by producing iron-chelating siderophores that can acquire otherwise sequestered ferric iron. Due to their capacity to enhance bacterial growth, siderophores are viewed as virulence factors (Buckling *et al.*^2007^). A wide body of research suggests that iron uptake strategy in Pseudomonads can be influenced by social context, because non-producers can exploit producers, and gain a fitness advantage (e.g. Griffin, West and Buckling 2004; Harrison and Buckling 2005, 2007; O’Brien, Rodrigues and Buckling 2013; Andersen *et al.*^2015^). However, most of these studies are limited to intraspecific interactions in spatially homogeneous environments (but see Luján *et al.*^2015^, and Harrison *et al*. 2017).

In the CF lung, many species compete for iron simultaneously, and this competition can indirectly shape iron-uptake strategies in *P. aeruginosa*. For instance, competition between *P. aeruginosa* and *B. cepacia* induces *P. aeruginosa* genes normally expressed under iron-limited conditions (including siderophores). This is because a *B. cepacia* siderophore, ornibactin (which *P. aeruginosa* cannot use), restricts iron availability to *P. aeruginosa* (Weaver and Kolter 2004). A similar phenomenon was observed using experimental evolution, whereby *P. aeruginosa* was evolved in the presence and absence of *S. aureus* (Harrison *et al.*^2008^). In this case, *P. aeruginosa* upregulated siderophore production in response to *S. aureus*, which acted as an iron competitor. Conversely, *P. aeruginosa* can obtain iron by lysing *S. aureus* cells (Mashburn *et al.*^2005^), although Harrison *et al.* (2008) suggest that this benefit depends on the degree of competition between the two strains. Interestingly, when multiple species compete for iron, subsequent iron limitation may also reduce the ability of *P. aeruginosa* to form biofilms (Singh *et al.*^2002^; O’May *et al.*^2009^). This is in line with what we observe in longitudinal studies of CF isolates, whereby iron becomes more available, and biofilms become more common over the course of infection (Hunter *et al.*^2013^; Tyrrell and Callaghan 2016; Winstanley, O’Brien and Brockhurst 2016). However, this correlation can be of course open to different interpretations.

There is some evidence to suggest that the requirement for siderophores is reduced in the later stages of infection, as freely available ferrous (Fe^2+^) tends to dominate over ferric iron (Hunter *et al.*^2013^). Furthermore, as host cells are damaged they release iron in the form of haem and haemoglobin, from which *P. aeruginosa* can sequester iron using the haem assimilation system (Has) and Phu (*Pseudomonas* haem uptake) systems. Indeed, over the course of chronic infections there is some evidence that siderophores are lost and replaced with haem utilisation (Marvig *et al.*^2014^). Finally, the role of pyocyanin in iron acquisition *per se* is poorly understood, although one study suggests that a different phenazine, PCA, assists in biofilm development by promoting ferrous iron (Wang *et al.*^2011^). Crucially, the role of various iron-uptake systems in shaping microbial communities may differ depending on the predominant form of acquisition. While siderophore sharing is generally species specific (Buckling *et al.*^2007^, but see Barber and Elde 2015), other acquisition mechanisms such as pyocyanin-mediated reduction is unlikely to be limited to conspecifics, and so understanding how they might be shaped by community interactions is not straightforward.

## CASE STUDIES

While the scope for interactions within the CF lung is clearly vast, we highlight interactions between *P. aeruginosa* and four commonly encountered species: the Gram-positive bacteria *S. aureus*, the *B. cepacia* complex (Gram-negative), a filamentous fungi (*A. fumigatus*) and *C. albicans* (a yeast) to display the breadth and diversity of interactions with *P. aeruginosa*.

### *P. aeruginosa* and *S. aureus*

*P. aeruginosa* and *S. aureus* display a striking negative correlation with one another as CF patients age (Cystic Fibrosis Foundation 2011), suggesting that *P. aeruginosa* can displace *S. aureus* in the later stages of infection. *P. aeruginosa* secretes a wealth of *S. aureus*-killing exoproducts, such as pyocyanin, elastase, protease, rhamnolipids, 4-hydroxy-2-alkylquinoline (HAQ) and 4-hydroxy-2-heptylquinoline-*N*-oxide (HQNO) (Mashburn *et al.*^2005^; Palmer *et al.*^2005^; Hoffman *et al.*^2006^; Mitchell *et al.*^2010^; Korgaonkar and Whiteley 2011; Cardozo *et al.*^2013^; Korgaonkar *et al.*^2013^; DeLeon *et al.*^2014^). *P. aeruginosa* can also harm *S. aureus* indirectly by manipulating the innate immunity of the host, such as inducing the production of *S. aureus*-killing phospholipase sPLA2-IIA by bronchial epithelial cells (Pernet *et al.*^2014^). This interaction between the host and *P. aeruginosa* enhances the clearance of *S. aureus* without significantly affecting the growth of *P. aeruginosa*. It is of course debatable whether the upregulation of sPLA2-IIA by *P. aeruginosa* has evolved as a competitor-killing mechanism, or if it is simply a response by the host to which *P. aeruginosa* is resistant. Nonetheless, sPLA2-IIA is the most potent known antibacterial enzyme in mammals, especially targeting Gram-positive bacteria, suggesting that interactions between *P. aeruginosa* and the host can shape bacterial communities more widely (Qu and Lehrer 1998; Nevalainen, Graham and Scott 2008). Finally, one recent study that experimentally evolved *P. aeruginosa* in the presence and absence of *S. aureus*, demonstrated that adaptation to *S. aureus* was mediated by inactivation of virulence-associated lipopolysaccharide (LPS) in *P. aeruginosa.* Crucially, this adaptation also conferred enhanced resistance to beta-lactam antibiotics, despite the fact that evolution took place in their absence (Tognon *et al.*^2017^).

Crucially, any counter adaptation by *S. aureus* to resist killing by *P. aeruginosa* can in turn shape the pathogenicity of *S. aureus*. Small colony variants of *S. aureus* (SCVs) arise by mutations in metabolic genes (Melter and Radojevic 2010) and experience reduced killing by *P. aeruginosa* HQNO's compared to their wild-type counterparts (Hoffman *et al.*^2006^; Biswas *et al.*^2009^; Filkins *et al.*^2015^). From a clinical perspective, SCVs display enhanced resistance to antibiotics (Wolter, Kotsiou and McCormack 1995), greater persistence (Hoffman *et al.*^2006^) and correlate with worsening symptoms in CF (Wolter *et al.*^2013^). Moreover, HQNO has been identified in CF patients harbouring *P. aeruginosa*, but not in uninfected individuals, suggesting that HQNO-mediated interactions between these two species have potential to directly influence disease progression (Hoffman *et al.*^2006^).

### *P. aeruginosa* and *A. fumigatus*

*Aspergillus fumigatus* is the most common fungus found in the CF airways (Nagano *et al.*^2007^; Pihet *et al.*^2009^), and its presence is associated with a diversity of clinical phenotypes ranging from no obvious respiratory decline, to *Aspergillus* bronchitis and bronchiectasis (Shoseyov *et al.*^2006^; Agarwal *et al.*^2013^; Chotirmall and McElavaney 2014). Infection tends to occur subsequent to *P. aeruginosa* colonisation, resulting in co-infections that trigger more severe clinical outcomes compared with *P. aeruginosa or A. fumigatus* alone (Amin *et al.*^2010^; Ferreira *et al.*^2015^; Reece *et al.*^2017^).

Several lines of evidence suggest that these two species interact extensively in the CF lung. *P. aeruginosa* has been classically viewed as inhibiting *A. fumigatus* growth by producing an array of phenazines which kill fungi at high concentrations (Kerr 1994; Moree *et al.*^2012^; Briard *et al.*^2015^). However, in the CF lung, the phenazines pyocyanin and phenazine-1-carboxylate have been found *in vivo* at concentrations in the range of 1–100 μM, which Briard *et al.* (2015) demonstrated to be subinhibitory against *A. fumigatus*. Furthermore, at these concentrations, these phenazines actually functioned as iron-reducing agents, liberating bioavailable iron and subsequently, fungal growth in iron-starved environments. Another phenazine, 1-HP, stimulated siderophore production in *A. fumigatus* and growth as a consequence Briard *et al.* (2015). Accordingly, there is a generally high percentage of Fe^2+^ once phenazine levels rise above 50 μM in sputum (Hunter *et al*. 2012, 2013). However, concentrations of phenazines may in reality vary within the lung, particularly in the lower respiratory tract where mucous is more concentrated. These findings may explain why *A. fumigatus* infections tend to occur after *P. aeruginosa* colonisation—*P. aeruginosa* creates an iron-rich environment in which *A. fumigatus* can thrive. However, an alternative explanation is that co-infection reduces the pro-inflammatory response exerted by the host, potentially enabling both strains to benefit (Reece *et al.*^2017^). Furthermore, damaged lungs *per se* may permit better colonisation by pathogens and increased virulence as a consequence.

### *P. aeruginosa* and *C. albicans*

Despite being the second most common fungus in CF, the role of *C. albicans* in CF is not fully understood. In practice, this means that a positive result for *C. albicans* in the clinic tells us little about patient prognosis. While invasive airway infections by *C. albicans* alone remain rare, its pathogenic effects may be experienced through interactions with other species. For instance, in mixed biofilms with *C. albicans, P. aeruginosa* upregulates its production of virulence-associated secretions such as pyoverdine, phenazines and rhamnolipids, relative to single-species biofilms (Hogan and Kolter 2002; Hogan, Vik and Kolter 2004; Cugini *et al.*^2007^; Gibson, Sood and Hogan 2009). Enhanced phenazine production by *P. aeruginosa* in turn upregulates *C. albicans* ethanol production, as the phenazines exert a switch from respiration to fermentation (Morales *et al.*^2013^). As mentioned previously, ethanol increases *P. aeruginosa* biofilm formation, resulting in mucoid phenotypes with enhanced growth rate (DeVault, Kimbara and Chakrabarty 1990; Morales *et al.*^2013^). This phenazine-mediated switch to fermentation in *C. albicans* may have consequences for microbiome diversity and composition. Ethanol has also been shown to enhance growth, virulence and biofilm formation in other lung pathogens such as *S. aureus* (Korem, Gov and Rosenberg 2010) and *Acinetobacter baumanii* (Nwugo *et al.*^2012^), although the exact mechanisms have not yet been elucidated. Ethanol is also an immunosuppressant, negatively influencing the host immune response (Greenberg *et al.*^1999^; Goral, Karavitis and Kovacs 2008). In a rat model system, ethanol inhibits lung clearance of *P. aeruginosa* by inhibiting macrophage recruitment (Greenberg *et al.*^1999^). Hence, ethanol may indirectly shape microbial communities by interfering with pathogen clearance. Another fermentation product, acetic acid, is also likely to reduce extracellular pH, which may favour the presence of low-pH-adapted microorganisms (Morales *et al.*^2013^).

Signalling can occur between these two species, influencing one another's gene expression and virulence (Fig. 2). The *P. aeruginosa* signal molecule 3-oxo-C12HSL promotes the yeast form of *C. albicans*, so that when levels of this signal drop (such as during chronic infection; Bjarnsholt *et al.*^2010^), the invasive, filamentous form of the fungus may be triggered (McAlester, O’Gara and Morrissey 2008). Conversely, *C. albicans* secrete the alcohol farnesol that suppresses the *P. aeruginosa* signal molecule PQS and consequently, pyocyanin production, while inducing quinolone and phenazine expression (Cugini *et al.*^2007^; Cugini, Morales and Hogan 2010). Finally, *C. albicans* can reduce the expression of the siderophores pyoverdine and pyochelin in *P. aeruginosa* leading to decreased virulence (Lopez-Medina *et al.*^2015^), although exact mechanisms have yet to be elucidated. Clearly, these interactions, and their effects on gene expression, are complex, and we are far from understanding how they will combine to influence host health.

### *P. aeruginosa* and *B. cepacia* complex

Secondary bacterial infections with the *B. cepacia* complex are associated with cepacia syndrome—a rapidly progressing and fatal pneumonia (Huang *et al.*^2001^; Lambiase *et al.*^2006^). Members of the *B. cepacia* complex form mucoid biofilms with *P. aeruginosa*, engaging in an intimate network of interactions, and possibly even exchanging genetic material (Eberl and Tümmler 2004).

Competition between these two species is rife. In one study that screened 66 *P. aeruginosa* and *B. cenocepacia* CF clinical isolates, 81% of *P. aeruginosa* and 57% of *B. cenocepacia* strains produced bacteriocin-like toxins, conferring inhibitory activity toward the other species (Bakkal *et al.*^2010^). Populations of *Burkholderia* have been found to invade populations of *P. aeruginosa* (Schwab *et al.*^2014^) and vice versa (Bragonzi *et al.*^2012^; Costello *et al.*^2014^), suggesting that the outcome of competition is highly context dependent. Interactions between these two species may also occur in more subtle ways: one class of signal molecules produced by *P. aeruginosa*, *N*-acyl homoserine lactones, can stimulate the production of siderophores, lipase and protease production in *Burkholderia* (McKenney *et al.*^1995^; Riedel *et al.*^2001^; Lewenza, Visser and Sokol 2002; Costello *et al.*^2014^). Moreover, alginate production by *P. aeruginosa* can aid *B. cenocepacia* survival by inhibiting the host immune response (Chattoraj *et al.*^2010^). Despite the role of both species as harmful pathogens, how their interactions may influence virulence is not well understood.

There has been a recent drive toward developing novel therapeutics using products secreted by naturally occurring competitors to target specific pathogens in not just CF (e.g. Brown *et al.*^2009^) but halitosis (Burton *et al.*^2006^) and *Clostridium difficile* (Rea *et al.*^2013^) infections. In particular, one *Burkholderia* bacteriocin named Tailocin has been proposed as a potential therapeutic with activity against *P. aeruginosa* (Yao *et al.*^2017^). In order to fully appreciate how *P. aeruginosa* populations will respond to these classes of drugs it is vital that we understand how the species involved interact naturally on both ecological and evolutionary timescales.

## OUTLOOK

There is tantalising evidence that interactions within and among species can alter virulence properties of *P. aeruginosa* in the short term and potentially shape the evolutionary trajectory of this pathogen in the long term. While our knowledge of how *P. aeruginosa* responds to other species individually is growing, the consequences of these interactions for virulence in a complex multispecies community remains unclear. Moreover, experimental evolution studies in complex environments containing the natural microbiota are required to decipher whether ecological responses drive selection for evolutionary change. On the one hand, multispecies infections may constrain the rate of evolutionary change if trade-offs are required to adapt to multiple species. On the other hand, increasing the number of interacting species may result in even more rapid evolution, as selection acts on increasing numbers of traits.

Understanding how virulence-associated secretions are shaped by the lung microbiome opens doors for novel therapeutic approaches already being exploited in gut microbiome research (Bakken *et al.*^2011^; Hamilton *et al.*^2012^; Lee *et al.*^2016^). For instance, an increased understanding of community dynamics could allow us to establish ‘infection-resistant’ communities to prevent initial colonisation of recognised pathogens or by replacing pathogens with commensal communities. This approach has already proved successful in treating *Clostridium difficile* gut infections with ‘healthy’ gut communities (Bakken *et al.*^2011^; Hamilton *et al.*^2012^; Lee *et al.*^2016^), and *Streptococcus mutans* dental caries with lactobacillus communities (Gungor, Kirzioglu and Kivanc 2015). Furthermore, through an increased understanding of the ecology of these lung communities, it may be possible to suppress *P. aeruginosa* indirectly by manipulating clinically relevant interactions.

However, there are many challenges. As a field, we are not clear on what a ‘normal’ or ‘healthy’ community might look like against a genetic background of CF. Furthermore, spatial structure in the lung, at both molecular and geographical scales, will impact the ability of colonising species to interact. However, the relevance of this structure for cell–cell interactions, as well as the extent to which sputum samples capture this structure, is unknown. Finally, the vast genotypic and phenotypic variation observed in *P. aeruginosa* populations from the same sputum sample (Mowat *et al.*^2011^; O’Brien *et al.*^2017^) suggests that in order to fully understand and characterise these fascinating populations, interactions should be considered on both a species and strain level. Clearly this is a hugely overwhelming task, but employing novel model systems that incorporate natural or semi-natural microbial communities allow us to make small steps toward achieving this goal (e.g. Harrison *et al.*^2014^).
